# Amniotic fluid embolism incidence, risk factors and outcomes: a review and recommendations

**DOI:** 10.1186/1471-2393-12-7

**Published:** 2012-02-10

**Authors:** Marian Knight, Cynthia Berg, Peter Brocklehurst, Michael Kramer, Gwyneth Lewis, Jeremy Oats, Christine L Roberts, Catherine Spong, Elizabeth Sullivan, Jos van Roosmalen, Joost Zwart

**Affiliations:** 1 National Perinatal Epidemiology Unit, University of Oxford, Oxford, UK; 2 Division of Reproductive Health, Centers for Disease Control and Prevention (CDC), Atlanta, GA, USA; 3 Department of Paediatrics and Department of Epidemiology and Biostatistics, McGill University Faculty of Medicine, Montreal, QC, Canada; 4 Consultative Council on Obstetric and Paediatric Mortality and Morbidity, Victoria, Australia; 5 The Kolling Institute of Medical Research, University of Sydney at Royal North Shore Hospital, Sydney, Australia; 6 Pregnancy and Perinatology Branch, Eunice Kennedy Shriver National Institute of Child Health and Human Development, NIH, Rockville, MD, USA; 7 Perinatal and Reproductive Epidemiology Research Unit, School of Women's and Children's Health, University of New South Wales, Sydney, Australia; 8 Department of Obstetrics, Leiden University Medical Center, Leiden, The Netherlands

## Abstract

**Background:**

Amniotic fluid embolism (AFE) is a rare but severe complication of pregnancy. A recent systematic review highlighted apparent differences in the incidence, with studies estimating the incidence of AFE to be more than three times higher in North America than Europe. The aim of this study was to examine population-based regional or national data from five high-resource countries in order to investigate incidence, risk factors and outcomes of AFE and to investigate whether any variation identified could be ascribed to methodological differences between the studies.

**Methods:**

We reviewed available data sources on the incidence of AFE in Australia, Canada, the Netherlands, the United Kingdom and the USA. Where information was available, the risk factors and outcomes of AFE were examined.

**Results:**

The reported incidence of AFE ranged from 1.9 cases per 100 000 maternities (UK) to 6.1 per 100 000 maternities (Australia). There was a clear distinction between rates estimated using different methodologies. The lowest estimated incidence rates were obtained through validated case identification (range 1.9-2.5 cases per 100 000 maternities); rates obtained from retrospective analysis of population discharge databases were significantly higher (range 5.5-6.1 per 100 000 admissions with delivery diagnosis). Older maternal age and induction of labour were consistently associated with AFE.

**Conclusions:**

Recommendation 1: Comparisons of AFE incidence estimates should be restricted to studies using similar methodology. The recommended approaches would be either population-based database studies using additional criteria to exclude false positive cases, or tailored data collection using existing specific population-based systems.

Recommendation 2: Comparisons of AFE incidence between and within countries would be facilitated by development of an agreed case definition and an agreed set of criteria to minimise inclusion of false positive cases for database studies.

Recommendation 3: Groups conducting detailed population-based studies on AFE should develop an agreed strategy to allow combined analysis of data obtained using consistent methodologies in order to identify potentially modifiable risk factors.

Recommendation 4: Future specific studies on AFE should aim to collect information on management and longer-term outcomes for both mothers and infants in order to guide best practice, counselling and service planning.

## Background

Amniotic fluid embolism (AFE) is a rare but severe complication of pregnancy. The rarity of the condition and the fact that AFE is a diagnosis of exclusion, make it particularly challenging to study and therefore difficult to obtain reliable information about incidence, risk factors, management and outcomes. Centre-based studies, because of the small population they cover, or because of the long historical period which has to be studied in order to identify a sufficient number of cases, rarely generate robust and reproducible results which can be generalised to today's obstetric populations. As AFE is infrequent, the most robust studies of the condition are population-based studies, ideally incorporating large numbers of pregnant women in order to have sufficient statistical power to generate stable incidence estimates and examine a range of risk factors to assess the independent risk associated with each. Multinational studies can enhance further the robustness, timeliness and hence utility of study results.

A recent systematic review highlighted apparent differences in the incidence of AFE in different studies [1], with estimates being more than three times higher in North America than Europe [2-6]. The aim of this study was to examine population-based regional or national data within five high resource countries in order to investigate incidence, risk factors and outcomes of AFE and to investigate whether any variation identified could be ascribed to methodological differences between the studies.

## Methods

We reviewed available data sources on the incidence of AFE in Australia, Canada, the Netherlands, the United Kingdom and the USA. Where information was available, data on the risk factors and outcomes of AFE were also reviewed. Because of variations in definitions of AFE used between countries and between data sources, we did not restrict our analysis to AFE defined using any one particular classification. The definitions and codes used to identify cases in each country are shown in table 1. Maternal death was defined throughout as death of a woman while pregnant or within 42 days of the termination of pregnancy.

**Table 1:** Definitions and codes used for AFE

Country	ICD-9 coding	ICD-10 Coding	Other definition used
Australia (NSW)	N/A	O88.1	1) if not fatal, the hospital record had to include a diagnosis of one or more of cardiac arrest, hypotension syndrome, respiratory distress, coagulation defects, coma and/or seizure, and an absence of other medical conditions or potential explanations of the symptoms and signs [1] and 2) where death was the outcome, AFE had to be listed as the cause of death.

Australia (Victoria)	N/A	O88.1	

Australia (national data)	673.1	O88.1	Review of maternal death by expert committee in conjunction with a cause of death of amniotic fluid embolism

Canada	673.1	O88.1	

Netherlands	N/A	N/A	- Reported as maternal mortality or severe maternal morbidity with AFE as diagnosis or in differential diagnosis - One or more of the following severe enough to require medical treatment: Hypotension (and/or cardiac arrest) Respiratory distress Disseminated intravascular coagulation Coma and/or seizures - Absence of any other clear medical explanation for the clinical course.

United Kingdom	N/A	N/A	***In the absence of any other clear cause*** **EITHER** Acute maternal collapse with one or more of the following features: Acute fetal compromise Cardiac arrest Cardiac rhythm problems Coagulopathy Hypotension Maternal hemorrhage Premonitory symptoms e.g. restlessness, numbness, agitation, tingling Seizure Shortness of breath *Excluding: *women with maternal hemorrhage as the first presenting feature, in whom there was no evidence of early coagulopathy or cardio-respiratory compromise **OR** Women in whom the diagnosis was made at post-mortem examination with the finding of fetal squames or hair in the lungs

USA	673.1	N/A	If not fatal, ICD-9CM code of 673.1. For fatal cases, coding indicating AFE as cause of death or major contributing factor to the death

### Data sources

#### Australia

Fatal cases were identified through four published triennial *Maternal Deaths in Australia reports *[7-10]. The Maternal Deaths in Australia reports are a compilation of confidential death reviews conducted at the jurisdictional level. Information for incident cases (fatal and non-fatal) was obtained from the National Hospital Morbidity Database (NHMD) [11] an administrative collection of hospital separations for the years 1994-2005. The NHMD is a collection of confidential summary records for admitted patients separated in public and private hospitals in Australia. The NHMD is based on data from the state and territory health authorities and is compiled nationally by the Australian Institute of Health and Welfare. Diagnoses and procedures were coded according to the International Classification of Diseases (ICD), tenth revision, Australian Modification (ICD10 AM) (ICD-9 prior to 1999). Records were selected based on a diagnosis of AFE using ICD9 Clinical Modification (CM) code of 637.10-637.14 with a birth code (V27.0-V27.2) or a code for care immediately following delivery (V24.0) or postpartum examination and care (V24.3). The ICD9 coding system includes a fifth digit (0-4) for obstetric codes that indicates the timing of the diagnosis of the condition relative to the birth; and for ICD-10AM O88.1 with Z37.0-Z37.9 used to flag a birth episode and Z39.01 to flag an immediate postpartum episode. Data were available for all jurisdictions in Australia, accounting for 100% of all women giving birth in hospital.

In New South Wales (NSW) data on incident cases (fatal and non-fatal) were obtained from the Admitted Patient Data Collection (APDC), a census of all hospital discharges from public and private hospitals, and linked to the Midwives Data Collection (MDC), a legislated surveillance system of all births in NSW completed by attending midwives and doctors [12]. Diagnoses and procedures obtained from the medical records for each hospitalisation are ICD-10-AM and the affiliated Australian Classification of Health Interventions. Data for 2001-2007 inclusive were examined. A previous validation study encountered only one case of AFE, and this was correctly coded [13].

Data for Victoria were derived from the Victorian Perinatal Data Collection (VPDC) which is completed by the birth attendant (usually the midwife) for each birth [14]. Diagnoses are coded according to ICD-10-AM (VPDC modification). Case validation was conducted through medical review of the Victorian Perinatal Data files of all cases identified. AFE diagnosis was confirmed if surviving mothers required admission to ICU, and/or had major blood transfusion of > 2 units, or pathology reports from autopsy were compatible with AFE.

#### Canada

As previously reported [4], information on incident cases (fatal and non-fatal) for Canada was based on all hospital deliveries as documented in the Discharge Abstract Database of the Canadian Institute for Health Information from 1991 to 2002, which records all deliveries in Canada with the exception of those in Quebec, Manitoba and Nova Scotia (thus approximately 70% of deliveries). All medical diagnoses were coded using the International Classification of Diseases (ICD-9 up to 2000, and a combination of ICD-9 and ICD-10 from 2001-2002), while procedures were coded using the Canadian Classification of Diagnostic, Therapeutic and Surgical Procedures (CCP) for 1991-2001 and the Canadian Classification of Interventions (CCI) for 2001-2.

#### The Netherlands

Fatal cases were identified from the Dutch Confidential enquiries into the causes of maternal mortality between 1983-1992 and 1993-2005 [15]. Incident cases (fatal and non-fatal) were identified through the LEMMoN study of severe maternal morbidity between 2004 and 2006 [16]. In each hospital in the Netherlands with a consultant-led maternity unit, a nominated reporting clinician notified cases on a monthly basis using a standard web-based reporting form. For both fatal and incident cases, specific information on diagnosis, characteristics of the case and management was collected using a standardised form in addition to copies of selected anonymised parts of the case record. These data were used for subsequent case validation by the committee.

#### United Kingdom

Fatal cases were identified through the United Kingdom (UK) confidential enquiries into maternal deaths between 2003 and 2008 [17,18].

UK data on incident cases of AFE (fatal and non-fatal) were obtained through the UK Obstetric Surveillance System (UKOSS) between February 2005 and January 2010 [3]. In each hospital in the UK with a consultant-led maternity unit, nominated reporting clinicians notified cases using a monthly reporting card. In response to a report of a case, clinicians were asked to complete a data collection form confirming the case definition, characteristics of the case, management and outcomes.

#### United States

Fatal cases were identified through the Pregnancy Mortality Surveillance System (PMSS) in the Division of Reproductive Health at the Centers for Disease Control and Prevention (CDC) [19]. Since 1986, PMSS has used matched vital records to identify deaths caused by pregnancy complications occurring during or within one year of the end of pregnancy.

Non-fatal and fatal incident cases were obtained from the Nationwide Inpatient Sample (NIS) for 1999-2008. The NIS is part of the Agency for Healthcare Research and Quality's Healthcare Cost and Utilization Project. The NIS [20] contains data on 5 to 8 million hospital stays and, with appropriate weighting, generates a nationally representative sample of inpatient hospital admissions. The database contains up to 15 diagnosis fields and 15 procedure fields; diagnoses and procedures are coded at the hospital at discharge using the ICD-9-CM. Except for age, the NIS does not collect individual demographic information nor does it report obstetrical characteristics for individual pregnancies except those that can be translated to ICD-9-CM codes. Delivery hospitalisations were identified using a previously published algorithm based on ICD-9-CM diagnosis and procedure codes and diagnosis-related group (DRG) codes [21].

### Statistical analyses

Incidence rates for AFE with 95% confidence intervals were calculated using as a denominator the number of maternities, deliveries or live births recorded regionally or nationally during each study period. Putative risk factors, identified from factors previously reported in the literature (maternal age, parity, smoking status, race/ethnicity, diabetes, multiple pregnancy, previous caesarean delivery, hypertensive disorders, placenta praevia, placental abruption, presentation at delivery, chorioamnionitis, polyhydramnios, induction of labour, mode of delivery, manual removal of placenta, macrosomia, gestational age at delivery) [1-4,22], were examined using univariable or full multivariable logistic regression analysis where possible and results presented as odds ratios (ORs) with 95% confidence intervals. Where individual level comparison group data were not available, associations were examined by comparing data on the proportion of cases with a particular risk factor with data on the proportion of the population of women giving birth with the same risk factor using risk ratios with 95% confidence intervals.

### Ethics Committee Approval

The UK AFE study was approved by the London Multi-centre Research Ethics Committee (ref 04/MRE02/46) and the linkage and use of New South Wales data was approved by the NSW Population and Health Services Research Ethics Committee (2006-06-011). The Dutch LEMMoN study was centrally approved by the medical ethics committee of the Leiden University Medical Centre (ref P04-020). No other permissions were required for use of the data presented.

## Results

### Incidence

The reported incidence of AFE ranged from 1.9 cases per 100 000 maternities (UK) to 6.1 per 100 000 maternities (Australia) (Table 2). There was a clear distinction between rates estimated using different methodologies. The lowest estimated incidence rates were obtained through validated prospective case identification (range 1.9-2.5 cases per 100 000 maternities); rates obtained from retrospective analysis of population discharge databases were significantly higher (range 5.5-6.1 per 100 000 admissions with delivery diagnosis), with an intermediate estimated incidence obtained from analysis of a regional discharge database in which additional required criteria (at least one of the cardinal symptoms with no other potential explanation) were used to exclude false positive cases (3.3 cases per 100 000 maternities). When the latter database was used without the additional selection criteria, the estimated incidence was 6.3/100,000.

**Table 2:** Incidence

Country	Years	Cases	AFE rate	95% CI
**Validated case identification**				
**UK**	2005-2010	72	1.9/100 000 maternities (deliveries ≥ 24 wks)	1.5-2.4
**Netherlands**	2004-2006	9	2.5/100 000 maternities (deliveries ≥ 24 wks)	1.3-4.8
**Victoria**	2000-2008	14	2.4/100 000 maternities (deliveries > 20 wks,400 g)	1.3-4.0
**Population Database with additional criteria to exclude false positive cases**				
**Australia (NSW)**	2001-2007	20	3.3/100 000 maternities (deliveries > 20 wks, 400 g)	1.9-4.7
**Population Database without additional criteria**				
**US [NIS]**	1999-2008	2226	5.5/100 000 admissions with delivery diagnosis	5.5-5.5
**Canada**	1991-2002	180	6.0/100 000 deliveries (> 20 wks 400 g)	5.3-7.1
**Australia**	1994-2005	185	6.1/100 000 admission a with a delivery diagnosis (deliveries > 20 wks, 400 g)	5.2-6.9

### Mortality and case fatality

Mortality ratios ranged from 0.4 per 100 000 live births in the Netherlands from 1993-2005 to 1.3 per 100 000 live births in the United States in 1997-2001 and 1.1 in Australia excluding Victoria in 1994-2005. Case-fatality rates ranged from 11 to 43%. There were no clear differences in estimates of maternal mortality due to AFE or case fatality rates due to AFE using any different methodology, noting the limited power to detect differences due to small numbers of fatal cases (Tables 3 and 4). The studies with the highest case fatality rates (NSW and Victoria) were conducted using case validation. Note, however, the wide confidence intervals surrounding all case fatality estimates due to the rarity of the condition and the small numbers involved.

**Table 3:** AFE Mortality ratios

Country	Years	Cases	Mortality	95% CI
**Validated case identification**				
**UK**	2003-2005	17	0.8 per 100 000 maternities	0.5-1.3
**UK**	2006-2008	13	0.6 per 100 000 maternities	0.3-1.0
**Netherlands**	1983-1992	2	0.1 per 100 000 live births	0.0-0.4
**Netherlands**	1993-2005	11	0.4 per 100 000 live births	0.2-0.8
**Australia (national)**	1994-2005	33	1.1 per 100 000 maternities	0.7-1.4
**Australia (Victoria)**	2000-2008	6	1.0 per 100 000 maternities	0.4-2.2
**Population Database with additional criteria to exclude false positive cases**				
**Australia (NSW)**	2001-2007	6	1.2 per 100 000 maternities	0.3-2.0
**Population Database without additional criteria**				
**US (PMSS)**	1991-1996	237	0.9 per 100 000 live births	*
**US (PMSS)**	1997-2001	256	1.3 per 100 000 live birth	*
**US (PMSS)**	2002-2005	171	1.0 per 100 000 live births	*
**Canada**	1991-2002	24	0.8 per 100 000 deliveries	0.5-1.2

*Based on data on all births therefore no confidence intervals quoted

**Table 4:** AFE case fatality

Country	Years	Case fatality rate	95% CI
**Validated case identification**			
**UK**	2005-2010	19%	11-30%
**Netherlands**	2004-2006	11%	3-45%
**Australia (Victoria)**	2000-2008	43%	18-71%
**Population Databases with additional criteria to exclude false positive cases**			
**Australia (NSW)**	2001-2007	35%	15-59%
**Population Databases without additional criteria**			
**US** **[PMSS+NIS]**	1993-1998	21%	18-22%
**US** **[PMSS+NIS]**	1999-2005	18%	17-21%
**Canada**	1991-2002	13%	8-19%
**Australia (national, NHMD)**	1994-2005	14%	9-19%

### Associated factors

The only factors consistently associated with AFE across all five countries were induction of labour and maternal age; although with both of these factors, in one country the association was not statistically significant. The association with age was not statistically significant in the Dutch data, this could be due to limited study power since all cases occurred in women who were aged 29 years or greater (Table 5). The methods of induction investigated varied. UK and Dutch data show a significant association between AFE and induction of labour (all methods); in the Canadian population, an association was observed with medical induction of labour, whereas in New South Wales there was only a statistically significant association with medical induction using vaginal prostaglandin. In the US study, induction of labour (all methods) was associated with raised odds (aOR 1.5), but this was not statistically significant. Where data were available, there were increased odds of AFE associated with placenta praevia and placental abruption. For all of the other factors examined (Tables 5, 6, 7, 8), the associations varied in the direction of effect and statistical significance, which may reflect small numbers and consequent lack of study power.

**Table 5:** Maternal factors associated with amniotic fluid embolism

	Australia (NSW)	Canada	Netherlands	UK	US
**Maternal factors**					

Age < 20 years	*	aOR 0.2 (0.1-0.96)	No cases	*	aOR 0.4 (0.2-0.9)

Age ≥ 35 years	RR 4.8 (2.0-12)	aOR 1.9 (1.4-2.7)	RR 2.4 (0.7-9.1)	aOR^≠ ^2.7 (1.4-5.1)	aOR 2.2 (1.5-2.1)

Multipara	RR 3.7 (0.9-8.1)	*	RR 6.6 (0.8-52.7)	aOR^≠ ^0.9 (0.5-1.7)	*

Smoked during pregnancy	RR 1.5 (0.5-4.6)	*	*	aOR^≠ ^0.9 (0.4-2.2)	*

Socioeconomic status	Disadvantaged (lowest quartile) RR 1.6 (0.6-4.0)	*	*	Routine, manual occupation or unemployed aOR^≠ ^0.5 (0.3-1.0)	*

Race/Ethnicity (Baseline group white or native born)	East Asian country of birth RR 2.4 (0.8-7.2)	*	Non-Western immigrants RR 1.3 (0.3-6.1)	Black or other ethnic minority group aOR^≠ ^1.2 (0.5-2.6)	African American aOR 2.4 (1.5-3.6) Other aOR 2.3 (1.5-3.6)

Diabetes (pregnancy and pre-pregnancy)	*	aOR 1.5 (0.6-3.8)	*	*	aOR 2.3 (0.6-9.2)

RR = Relative Risk, aOR = adjusted Odds Ratio

^≠ ^Adjusted for age, parity, smoking status, socioeconomic status, ethnicity, bmi, multiple pregnancy, placenta praevia or abruption, labour induction and postdates

* No data or no accurate or limited data

**Table 6:** Pregnancy factors associated with amniotic fluid embolism

	Australia (NSW)	Canada	Netherlands	UK	US
**Pregnancy factors**					

Multiple pregnancy	*	OR 2.5 (0.9-6.2)	RR 7.1 (0.9-56.5)	aOR^≠ ^5.3 (1.2-23.0)	*

Previous caesarean	RR 1.7 (0.5-6.0)	aOR 0.8 (0.5-1.2)	*	*	aOR 0.9 (0.6-1.3)

Hypertensive disorders	Any hypertensive disorder RR 1.3 (0.3-5.5) Pre-existing hypertension RR 9.5 (2.2-41)	Eclampsia aOR 11.5 (2.8-48.6) Pre-eclampsia aOR 1.4 (0.8-2.5)	*	*	Eclampsia aOR 29.1 (7.1-119) Pre-eclampsia aOR 7.3 (4.3-12.5)

Placenta praevia	RR 10.5 (1.4-79)	Praevia or abruption aOR 3.5 (2.3-5.5)	*	aOR^≠ ^15.6 (2.5-98.8)	aOR 30.4 (15.4-60)

Placental abruption	RR 13.3 (1.8-100)		*	aOR^≠ ^17.3 (1-304.1)	aOR 8.0 (4.0-15.9)

Non-vertex at delivery	RR 6.8 (2.4-18.8)	*	RR 2.4 (0.3-19.4)	*	*

Chorioamnionitis	No cases	aOR 1.4 (0.6-3.2)	*	*	aOR 1.6 (0.7-3.4

Polyhydramnios	*	aOR 3.0 (1.2-7.3)	*	*	*

RR = Relative Risk, aOR = adjusted Odds Ratio

^≠ ^Adjusted for age, parity, smoking status, socioeconomic status, ethnicity, BMI, multiple pregnancy, placenta praevia or abruption, labour induction and postdates

* No data or no accurate or limited data

**Table 7:** Associations between induction of labour and amniotic fluid embolism

	Australia (NSW)	Canada	Netherlands	UK	US
**Induction of labour**					

Induction of labour (all methods)	*	*	RR 5.6 (1.5-20.9)	aOR^≠ ^3.5 (1.9-6.7)	aOR 1.5 (0.9-2.3)

Specific methods of induction					

Any medical induction of labour	RR 1.9 (0.8-4.9)	aOR 1.8 (1.3-2.7)	*		

Surgical induction of labour/artificial rupture of membranes	RR 1.4 (0.6-3.5)	1.0 (0.6-1.8)	*	*	*

Vaginal prostaglandin (PG) E2 (induction)	RR 3.4 (1.3-9.0)	*	*	*	*

Any oxytocin	RR 0.8 (0.3-2.3)	*	*	*	*

Induction with both PG+oxytocin	RR 2.1 (0.5.-9.1)	*	*	*	*

RR = Relative Risk, aOR = adjusted Odds Ratio

^≠ ^Adjusted for age, parity, smoking status, socioeconomic status, ethnicity, bmi, multiple pregnancy, placenta praevia or abruption, labour induction and postdates

* No data or no accurate or limited data

**Table 8:** Delivery factors associated with amniotic fluid embolism

	Australia (NSW)	Canada	Netherlands	UK	US
**Delivery**					

Mode of delivery					

(baseline group normal vaginal delivery)	caesarean section After labour RR 48.5 (6.1-380) Without labour RR 8.1 (0.7-89)	caesarean section Cephalic aOR 12.5 (7.9-19.9) Non-cephalic aOR 8.6 (4.3-17.4)	caesarean section RR 2.2 (0.5-11.1)	**Cases occurring post delivery only** caesarean section aOR^≈^ 20.3 (4.3-95.3)	caesarean section aOR 5.7 (3.7-8.7)

	instrumental RR 36.0 (4.4-300)	Forceps aOR 5.9 (3.4-10.3) Vacuum aOR 2.9 (1.6-5.3)	Vacuum RR 1.5 (0.2-12.3)	Forceps or vacuum aOR^≈^ 11.6 (1.7-79.8)	Forceps aOR 4.3 (1.9-7.6) Vacuum aOR 1.9 (1.0-3.7)

	vaginal breech birth RR 151 (9.4-2400)			**All cases** Caesarean section aOR^¥ ^23.3 (7.8-69.7) Forceps or vacumm aOR^¥ ^8.9 (2.0-39.5)	

Manual removal of placenta (vaginal births)	RR 19.4 (3.9-96)	*	*	*	*

Macrosomia	*	aOR 1.6 (0.9-3.0)	*	OR 2.4 (0.7-8.1)	*

					

Gestational age	< 37 weeks RR 1.9 (0.4-8.6) 37-40 weeks 1.0 (referent) ≥ 41 weeks RR 1.7 (0.6-4.8)	> 42 weeks aOR 1.4 (0.9-2.3)	< 37 weeks RR 9.7 (2.3-40.8) > 42 weeks RR 3.2 (0.4-25.8)	≥ 41 weeks aOR^≠ ^0.4 (0.2-1)	*

RR = Relative Risk, aOR = adjusted Odds Ratio

* No data or no accurate or limited data

≈ Adjusted for age, parity, smoking status, socioeconomic status, ethnicity, bmi, placenta praevia, placenta abruption, labour induction, postdates and mode of delivery

^¥ ^Adjusted for age, parity, smoking status, socioeconomic status, ethnicity, bmi, multiple pregnancy, placenta praevia, placenta abruption, labour induction, postdates and mode of delivery

Other associations were noted with mode of delivery: forceps/vacuum and caesarean section, although these are challenging to interpret since information on the timing of delivery in relation to the AFE was not available for all of the data sources. In the UK data, where timing of the event and delivery was available, there was a statistically significant association noted with caesarean section delivery when the AFE occurred after delivery. There was no association with forceps or vacuum delivery, although the small number of women with AFE who had operative vaginal deliveries means there is limited statistical power to examine this association. Of note, eclampsia, a condition which may form one of the differential diagnoses for AFE, was strongly associated with AFE in the Canadian and US studies which did not use additional criteria to exclude false positive cases.

Very limited data were available on factors associated with fatality (Table 9); there were no factors consistently associated with fatality across all of the countries.

**Table 9:** Factors associated with fatality amongst AFE cases (risk ratio (RR) unless indicated)

Condition or procedure	Australia (NSW)	Canada	UK	US
Age ≥ 35 years	1.1 (0.5-2.4)	*	aOR^≠ ^2.8 (0.5-14.2)	*

Multipara	1.1 (0.7-1.7)	*	aOR^≠ ^0.9 (0.2-4.2)	*

Smoked during pregnancy	1.4 (0.3-6.5)	*	*	

Socioeconomic status	Disadvantaged (lowest quartile) 1.4 (0.4-4.5)	*	Routine, manual occupation or unemployed aOR^≠ ^1.2 (0.3-5.6)	Income $35,000+ aOR 0.3 (0.1-1.0)

Race/ethnicity			Black or other ethnic minority group vs white aOR^≠ ^6.3 (1.1-34.9)	

Previous caesarean	1.1 (0.1-9.7)	*	*	aOR 1.1 (0.3-3.9)

Induction		OR 3.5 (1.5-8.4)	aOR^≠ ^1.9 (0.4-9.2)	aOR 0.7 (0.1-3.3)

Medical induction of labour	1.6 (0.5-5.1)	*	*	

PG+Oxytocin	2.2 (0.2-29)	*	*	*

General anaesthesia	1.6 (0.8-2.9)			

Epidural anaesthesia	2.2 (0.2-29)			

Mode of delivery	Forceps/vacuum 0.4 (0.1-2.9) CS 1.8 (0.9-3.6)	*		Forceps aOR 1.7 (0.2-14.7) Vacuum aOR 0.7 (0.1-5.0) CS aOR 0.8 (0.2-2.8)

* No data or no accurate or limited data

^≠ ^Adjusted for age, parity, socioeconomic status, ethnicity and labour induction

### Outcomes

For the majority of sources used, there was very limited information on maternal outcomes other than death. Cerebral injury was noted in 6% of women with AFE in the UK, and cerebral infarction occurred in 20% of women with AFE in New South Wales. Data were similarly limited on fetal and infant outcomes. Eight of 21 infants (38%) born to mothers with AFE in the Netherlands were stillborn or died in the neonatal period; the figure was 5 of 75 infants (7%) in the UK and 6 of 19 (32%) in NSW.

## Discussion

### Incidence of AFE

This analysis demonstrates differences in the reported incidence of AFE in high-resource countries; the reported incidences vary according to the study methodology. Incidence estimates generated from analysis of population databases without additional criteria to exclude false positive cases produce more than double the estimates generated from analyses using specific validated case identification; an estimate from a population database analysis with additional criteria to exclude false positive cases was compatible with the estimates from the validated case identification studies. Comparison of the AFE incidence using a population database with and without criteria to exclude false positive cases also found this pattern, with the estimated incidence from the unselected population database double that when the additional criteria were used. This suggests that the noted European-North American differences in incidence [1] are also likely to be explained by the differences in methodology used, and may not represent a true difference in incidence between the countries.

It is difficult to determine which of these estimates is likely to represent a value closest to the "true" incidence of AFE. The apparently lower case fatality amongst cases identified through database studies supports the argument that these studies include a number of false positive cases. The observed association with conditions which may form part of the differential diagnosis of amniotic fluid embolism, for example eclampsia, also supports this hypothesis. Conversely, the higher case fatality observed in studies with case validation may simply indicate that these studies identify more severe cases. Database studies without case validation may thus have high sensitivity but conversely low positive predictive value due to the inclusion of a number of false positive cases, whereas studies with case validation may have lower sensitivity, due to lower case ascertainment and false negative cases, but higher positive predictive value. Perhaps the gold standard would be a database study with subsequent case validation through examination of the medical records. Such an approach, however, in the context of a rare condition such as AFE is difficult due to the large number of individual centres at which medical records would need to be accessed. All studies are complicated by the additional fact that AFE is a diagnosis of exclusion; internationally accepted diagnostic criteria for non-fatal cases do not exist, and there is a place for development of such criteria, using as a model, for example, the criteria used within the UK [3]. Differences in the case definitions used are likely to add to the observed variation in incidence estimates.

Thus the optimal approach to investigating AFE may be dependent on the purpose of the study, since each of the practical approaches has both advantages and disadvantages. Population-based database studies, where suitable sources of information exist, are likely to be the most economical option for studying the condition; however, there is likely to be a degree of inclusion of false positive cases, over-estimation of incidence and thus non-uniformity of the cases examined. The use of additional criteria to exclude false positive cases within database studies, the approach taken in New South Wales [22], may overcome some of these issues. However, without review of the medical records, this approach may exclude true cases that have been poorly coded. Database studies are also limited by a restricted number of available data items; according to the purpose of the original data collection, extremely limited information on management is available, and temporality of events, for example in relation to whether the AFE occurred before or after birth, cannot be studied. For the purposes of examining risk factors and management in detail, studies with specific prospective data collection methods may therefore be the best approach. Such studies are facilitated by available collaborations designed specifically to study rare pregnancy disorders, such as the approach taken by UKOSS [3], the Australasian Maternity Outcomes Surveillance System (AMOSS) and other members of the International Network of Obstetric Survey Systems (INOSS) [23].

**Recommendation 1: **Comparisons of AFE incidence estimates should be restricted to studies using similar methodology. Depending on the available resources and research questions, the recommended approaches would be either population-based database studies using additional criteria to exclude false positive cases, or tailored data collection using existing population-based systems specifically designed to facilitate study of rare pregnancy conditions.

**Recommendation 2: **Comparisons of AFE incidence between and within countries would be facilitated by development of an agreed case definition, particularly for non-fatal cases, and an agreed set of criteria to minimise inclusion of false positive cases for studies conducted using population-based databases.

### Associated factors

We identified very little consistency among countries in the factors associated with occurrence of AFE and no factors consistently associated with fatality from AFE. Maternal age appeared to be associated with the occurrence of AFE in all populations examined, and this may therefore represent a true association, although the mechanisms by which older maternal age predisposes to AFE remain hypothetical and may include disruption of the uterine vasculature or minor degrees of abnormal placental invasion. The available information on placental abnormalities suggests that placenta praevia and placental abruption substantially increase (3-10 times) the risk of AFE. Given the differing methodologies and therefore differing biases due to potential inclusion of false positive cases or selection of more severe cases, other reported associations are very difficult to interpret. One of the most widely reported associations, with induction of labour, was noted to be statistically significant in some studies (UK, Netherlands), with only certain induction methods (Australia, Canada) or the association was not statistically significant, although the estimated odds ratio was compatible with those reported in most other studies (US) [2]. This illustrates the difficulties of comparing data across studies with differing case identification processes and definitions and varying levels of detail about individual risk factors.

With other factors, such as mode of delivery, issues concerning the timing of the event make the data difficult to interpret. The data we examined were often limited, frequently because the timing of the AFE in relation to the delivery was not available, and hence we were unable to identify cases in which a caesarean or operative vaginal delivery was a cause and not a consequence of the AFE. In the studies where timing was available, small numbers of women in certain subgroups, for example the number of cases undergoing operative vaginal delivery, limited statistical power. It is important to note, however, in the context of rising caesarean delivery rates worldwide, that there was a significant association with caesarean delivery in the one country (UK) in which we were able to investigate specifically cases where the AFE occurred after delivery. Analysis of pooled international data, obtained using consistent methodologies with agreed definitions and case validation, would provide more reliable information on these associated factors and hence provide the potential to develop appropriate preventive strategies which would otherwise be limited.

**Recommendation 3: **Groups conducting detailed population-based studies on AFE should develop an agreed strategy to allow combined analysis of data obtained using consistent methodologies in order to identify potentially modifiable risk factors.

### Outcomes

There were no clear differences in maternal mortality ratios due to AFE between countries or by study methodology. Information on other outcomes for women was very limited, although data on cerebral injury [3] suggest that AFE may have a significant impact in the long term outcomes of survivors, and this requires further investigation. None of the data sets included information on long-term maternal outcome after non-fatal AFE. Data on infant outcomes were only available for sources which can link data on the mother and infant or from studies which collect specific data. Although limited, the available data suggest the perinatal death rate associated with AFE is high (7-38%). Similarly, we were unable to investigate any potential relationship between management and outcomes because of extremely limited data. Further international collaborative studies using specific data collection would allow for more detailed investigation of the outcomes for mother and infant, over both short and longer term, and any relationship with management.

**Recommendation 4: **Future specific studies on AFE should aim to collect information on management and longer-term outcomes for both mothers and infants in order to guide best practice, counselling and service planning.

### Strengths and limitations of this analysis

This analysis used data from five high resource countries, and the results are thus generalizable only to countries with similar resource settings. As discussed above, data on AFE were obtained using different study methodologies, and therefore this limits the comparability of some of the results. AFE is a rare condition, and all studies have limited power to detect true associations as statistically significant. We hope that this may be addressed in the future by further international collaborative studies, an approach that we would advocate for research into all rare conditions in pregnancy.

## Conclusions

This analysis has highlighted the benefits of detailed comparison of AFE incidence and ascertainment methods from different population-based studies and has identified a number of difficulties with making direct international comparisons. The study methodology impacts on estimates of disease incidence and case fatality, and may also account for inconsistencies in reported risk factors. There is a need for consistent study methodologies, including agreed case definition and case validation criteria. The use of such unified methodologies will allow for valid international comparisons of incidence in the future, and may permit pooling of international data to provide more reliable information on associated factors, management and outcomes, thus allowing for development of preventive and treatment strategies to improve outcomes of this rare but serious condition.

## Competing interests

The authors declare that they have no competing interests.

## Authors' contributions

MKn conceived the study, participated in the workshop, presented data, contributed to discussions and drafted the manuscript/paper. CB participated in the workshop, presented data, contributed to discussions and participated in revising the manuscript. PB participated in the workshop, presented data, contributed to discussions and participated in revising the manuscript. MKr participated in the workshop, presented data, contributed to discussions and participated in revising the manuscript. GL participated in the workshop, presented data, contributed to discussions and participated in revising the manuscript. JO participated in the workshop, presented data, contributed to discussions and participated in revising the manuscript. CR participated in the workshop, presented data, contributed to discussions and participated in revising the manuscript. CS participated in the workshop, presented data, contributed to discussions and participated in revising the manuscript. ES participated in the workshop, presented data, contributed to discussions and participated in revising the manuscript. JvR participated in the workshop, presented data, contributed to discussions and participated in revising the manuscript. JZ participated in the workshop, presented data, contributed to discussions and participated in revising the manuscript. All authors read and approved the final manuscript.

## Pre-publication history

The pre-publication history for this paper can be accessed here:

http://www.biomedcentral.com/1471-2393/12/7/prepub

## References

[B1] Conde-Agudelo A Romero R Amniotic fluid embolism: an evidence-based review Am J Obstet Gynecol 2009 201 5 445 e441-413 19879393 10.1016/j.ajog.2009.04.052 PMC3401570

[B2] Abenhaim HA Azoulay L Kramer MS Leduc L Incidence and risk factors of amniotic fluid embolisms: a population-based study on 3 million births in the United States Am J Obstet Gynecol 2008 199 49 e1 e8 18295171 10.1016/j.ajog.2007.11.061

[B3] Knight M Tuffnell D Brocklehurst P Spark P Kurinczuk JJ Incidence and risk factors for amniotic-fluid embolism Obstet Gynecol 2010 115 5 910 917 10.1097/AOG.0b013e3181d9f629 20410762

[B4] Kramer MS Rouleau J Baskett TF Joseph KS Amniotic-fluid embolism and medical induction of labour: a retrospective, population-based cohort study Lancet 2006 368 1444 1448 10.1016/S0140-6736(06)69607-4 17055946

[B5] Pallasmaa N Ekblad U Gissler M Severe maternal morbidity and the mode of delivery Acta Obstet Gynecol Scand 2008 87 6 662 668 10.1080/00016340802108763 18568466

[B6] Samuelsson E Hellgren M Hogberg U Pregnancy-related deaths due to pulmonary embolism in Sweden Acta Obstet Gynecol Scand 2007 86 4 435 443 10.1080/00016340701207500 17486465

[B7] Sullivan E Hall B King J Maternal deaths in Australia 2003-2005 2008 Canberra: Australian Institute of Health and Welfare

[B8] Sullivan E King J Maternal deaths in Australia 2000-2002 2006 Canberra: Australian Institute of Health and Welfare vol. Maternal Deaths Series no. 2. Cat. no. PER 32

[B9] Slaytor EK Sullivan E King J Maternal deaths in Australia 1997-1999 2004 Canberra: Australian Institute of Health and Welfare vol. Maternal Deaths Series no. 1. Cat. no. PER 32 10.5694/j.1326-5377.2004.tb06361.x 15487953

[B10] NHMRC Report on Maternal Deaths in Australia, 1994-96 2001 Canberra: Commonwealth of Australia

[B11] National hospital morbidity database (NHMD) http://www.aihw.gov.au/national-hospital-morbidity-database/

[B12] L Ford JB Roberts CL Taylor LK Characteristics of unmatched maternal and baby records in linked birth records and hospital discharge data Paediatr Perinat Epidemiol 2006 20 4 329 337 10.1111/j.1365-3016.2006.00715.x 16879505

[B13] Roberts CL Cameron CA Bell JC Algert CS Morris JM Measuring maternal morbidity in routinely collected health data: development and validation of a maternal morbidity outcome indicator Med Care 2008 46 8 786 794 10.1097/MLR.0b013e318178eae4 18665058

[B14] Victorian Perinatal Data Collection (VPDC) http://www.health.vic.gov.au/ccopmm/vpdc/index.htm

[B15] Schutte JM Steegers EA Schuitemaker NW Santema JG de Boer K Pel M Vermeulen G Visser W van Roosmalen J Rise in maternal mortality in the Netherlands BJOG 2010 117 4 399 406 10.1111/j.1471-0528.2009.02382.x 19943828

[B16] Zwart JJ Richters JM Ory F de Vries JI Bloemenkamp KW van Roosmalen J Severe maternal morbidity during pregnancy, delivery and puerperium in the Netherlands: a nationwide population-based study of 371,000 pregnancies BJOG 2008 115 7 842 850 10.1111/j.1471-0528.2008.01713.x 18485162

[B17] Lewis GE The Confidential Enquiry into Maternal and Child Health (CEMACH). Saving Mothers Lives: reviewing maternal deaths to make childhood safer - 2003-2005 2007 London: CEMACH

[B18] Lewis GE The Centre for Maternal and Child Enquiries (CMACE). Saving Mothers Lives: reviewing maternal deaths to make childhood safer - 2006-2008 2011 London: CMACE

[B19] Chang J Elam-Evans LD Berg CJ Herndon J Flowers L Seed KA Syverson CJ Pregnancy-related mortality surveillance--United States, 1991--1999 MMWR Surveill Summ 2003 52 2 1 8 12825542

[B20] Overview of the Nationwide Inpatient Sample (NIS) http://www.hcup-us.ahrq.gov/nisoverview.jsp

[B21] Kuklina EV Whiteman MK Hillis SD Jamieson DJ Meikle SF Posner SF Marchbanks PA An enhanced method for identifying obstetric deliveries: implications for estimating maternal morbidity Matern Child Health J 2008 12 4 469 477 10.1007/s10995-007-0256-6 17690963

[B22] Roberts CL Algert CS Knight M Morris JM Amniotic fluid embolism in an Australian population-based cohort BJOG 2010 117 11 1417 1421 10.1111/j.1471-0528.2010.02656.x 21126320

[B23] The International Network of Obstetric Survey Systems (INOSS) Retrieved 19/08/2010, 2010, from https://www.npeu.ox.ac.uk/inoss

